# Adipose-Derived Stem Cells Primed with Paclitaxel Inhibit Ovarian Cancer Spheroid Growth and Overcome Paclitaxel Resistance

**DOI:** 10.3390/pharmaceutics12050401

**Published:** 2020-04-27

**Authors:** Cinzia Borghese, Naike Casagrande, Giuseppe Corona, Donatella Aldinucci

**Affiliations:** 1 Molecular Oncology Unit, Centro di Riferimento Oncologico di Aviano (CRO), IRCCS, Aviano, 33081 Pordenone, Italy; cpborghese@cro.it (C.B.); naike.casagrande@cro.it (N.C.); 2 Immunopathology and Cancer Biomarkers Unit, Centro di Riferimento Oncologico di Aviano (CRO) IRCCS, 33081 Aviano, Italy; giuseppe.corona@cro.it

**Keywords:** adipose-derived stem cells, ovarian cancer spheroids, anticancer chemotherapy, drug delivery, paclitaxel resistance

## Abstract

Adipose-derived stem cells (ADSCs) primed with paclitaxel (PTX) are now hypothesized to represent a potential Trojan horse to vehicle and deliver PTX into tumors. We analyzed the anticancer activity of PTX released by ADSCs primed with PTX (PTX-ADSCs) (~20 ng/mL) in a panel of ovarian cancer (OvCa) cells sensitive or resistant to PTX. We used two (2D) and three dimensional (3D) in vitro models (multicellular tumor spheroids, MCTSs, and heterospheroids) to mimic tumor growth in ascites. The coculture of OvCa cells with PTX-ADSCs inhibited cell viability in 2D models and in 3D heterospheroids (SKOV3-MCTSs plus PTX-ADSCs) and counteracted PTX-resistance in Kuramochi cells. The cytotoxic effects of free PTX and of equivalent amounts of PTX secreted in PTX-ADSC-conditioned medium (CM) were compared. PTX-ADSC-CM decreased OvCa cell proliferation, was more active than free PTX and counteracted PTX-resistance in Kuramochi cells (6.0-fold decrease in the IC50 values). Cells cultivated as 3D aggregated MCTSs were more resistant to PTX than 2D cultivation. PTX-ADSC-CM (equivalent-PTX) was more active than PTX in MCTSs and counteracted PTX-resistance in all cell lines. PTX-ADSC-CM also inhibited OvCa-MCTS dissemination on collagen-coated wells. In conclusion, PTX-ADSCs and PTX-MSCs-CM may represent a new option with which to overcome PTX-resistance in OvCa.

## 1. Introduction

Improving the delivery of cancer therapies to tumor sites is fundamental to decreasing their negative side effects. In this context, mesenchymal stem cells (MSCs) have been proposed as cellular vehicles for targeted cancer therapies, thanks to their tumor homing properties. MSCs are present in many different mammalian tissues (adipose tissue, bone marrow, skin, umbilical cord blood, placenta, etc.) and are easy to isolate and expand [1].

Based on these characteristics, several laboratories set up models of engineered MSCs as vehicles for anticancer molecules, such as interferons, growth factors, chemokines and drugs [2,3]. Alternatively, the ability of MSCs to incorporate and release drugs in the conditioned medium (CM) or secretome was used against cancer cells [4,5,6].

MSCs from different sources, including adipose tissues (adipose-derived stem cells, ADSCs), without any genetic manipulation, acquire strong anti-tumor activity after priming with the chemotherapeutic drug paclitaxel (PTX) through their capacity to uptake and release drugs in CM [3,6,7,8]. Furthermore, CM from MSCs has been recently reported to be a possible alternative to MSCs for therapeutic purposes [4,5,6]; it contains released drugs, including PTX, not only as free molecules but also associated to extracellular vesicles (EVs) [5,9]. EVs are cell-derived membranous structures which are shed from the plasma membrane, and transport biologically active molecules to local or distant sites [10]. EVs can carry lipophilic and hydrophilic drugs, representing an additional manner for anticancer drug delivery [9,11].

Epithelial ovarian cancer (OvCa) is the ninth most prevalent cancer and the fifth most common cause of cancer-related death in women [7]. The standard postoperative chemotherapy for OvCa is a combination therapy including cisplatin and taxanes [12]. Most patients with OvCa are responsive to chemotherapy at first; however, toxicity and acquired resistance to cisplatin or taxanes represent still the major obstacles to improve prognosis [7,13].

OvCa dissemination is associated with malignant ascites, which is present in a third of patients at diagnosis and almost in all patients at recurrence, and it is considered a major source of chemo-resistance, recurrence and mortality. Malignant ascites contain tumor cells as single cells, or more commonly, as aggregates of multicellular tumor spheroids (MCTSs). MCTSs are more resistant to anticancer drugs and are considered bona fide metastatic units that can attach to the mesothelium and invade the extracellular matrix of distant abdominal sites during dissemination [14].

Considering the peculiar intraperitoneal tumor growth and dissemination of OvCa, the use of ADSCs primed with PTX may not represent an efficacious strategy to treating drug resistant MCTSs. However, our working goal is to test the potential of PTX-primed ADSCs cells or their conditioned medium on OvCa MCTSs models to overcome drug resistance.

Using monolayer cultures (2D model) and multicellular spheroids (3D model) from OvCa cells, we showed that PTX secreted by PTX-ADSCs, at equivalent concentrations, was more active than free PTX, overcame PTX resistance and inhibited both migration and dissemination of OvCa cells.

## 2. Material and Methods

### 2.1. Drugs

Paclitaxel (PTX) (ACTAVIS) was a surplus drug from the clinical pharmacy of CRO Aviano.

### 2.2. Cell Culture

Ovarian cancer cell lines A2780 and A2780cis were from Sigma-Aldrich; SKOV3 (HTB-77) and COV318 were obtained from the American Type Culture Collection (ATCC); OVCAR5 (NIH) cells were provided by Dr. Baldassarre (CRO, Aviano, Italy); Kuramochi cells (JCRB0098), resistant to paclitaxel [15], were from JCRB Cell Bank; and OVCAR8 cells were from the National Cancer Institute Developmental Therapeutics Program Tumor Repository. All cell lines were routinely tested for mycoplasma, with negative results, and authenticated in our laboratory using PowerPlex 16 HS System (Promega, Madison, WI, USA) and GeneMapper ID version 3.2.1 to identify DNA short tandem repeats. ADSCs (Lonza) were maintained in Mesenchymal-Stem-Cell Growth Medium Bulletkit MSCGM (Lonza, Verviers, Belgium). Ovarian cancer cell lines were maintained in RPMI-1640 medium (Sigma-Aldrich Co., St. Louis, MO, USA) containing 10% heat-inactivated FBS, 100 U/mL penicillin and streptomycin (complete medium), at 37 °C in a 5% CO2 atmosphere. A2780cis cells were maintained in 1 µM cisplatin and cultured without the drug for 72 h before use in cellular assays. To obtain 3D-multicellular tumor spheroids (MCTSs), plates were coated twice with 20 mg/mL of poly(2-hydroxyethyl methacrylate) (poly-HEMA; Sigma) in 95% ethanol and washed once with PBS before cell seeding (2.0 × 10^4^ cells in 24-well).

### 2.3. PTX-Uptake and Release by ADSCs in Conditioned Medium (CM)

Priming of ADSCs was performed as previously described [3,16]. Briefly, sub-confluent cultures of ADSCs were exposed to 2 µg/mL PTX for 24 h. Then cells were washed twice with PBS, detached and then seeded in a new flask. After 24 h of culture, CM was collected and PTX-primed ADSCs (PTX-ADSCs) were used to evaluate cell cycle phases and migration. Three separate samples from PTX-ADSCs were pooled together to analyze the concentration of released PTX [17] by LC-MS/MS, as described in Supplementary Materials; then PTX-ADSC-CM was used to perform experiments. ADSC-CM obtained in the same experimental conditions but without PTX loading was used as control.

### 2.4. Cytotoxicity, Proliferation and Cell Cycles of ADSCs and PTX-ADSCs

ADSCs were cultured in triplicate in complete medium in flat bottom 96 well plates in the presence of different PTX concentrations (0–10 μg/mL). The cytotoxic activity (1.0 × 10^4^ cells) and the effect on proliferation (2.0 × 10^3^ cells) were evaluated using the 3-(4,5-dimethylthiazol-2-yl)-2,5-diphenyltetrazolium bromide (MTT) assay after 24 h and 7 days of treatment, respectively. To analyze the distribution of cells in the various phases of the cell cycle, ADSCs and PTX-ADSCs were fixed in cold 70% ethanol for 15 min and stained with PI solution (50 µg/mL PI, 0.1% NP-40, 100 µg/mL PureLink RNase A, 0.1% sodium citrate). After 1 h, cells were analyzed by flow cytometry. The distribution of cells in different cell cycle phases was quantified using ModFit LT 4.0 software. Half maximal inhibitory concentrations (IC50) were calculated using CalcuSyn software, v2.1 (Biosoft, Ferguson, MO, USA) [18].

### 2.5. Fluorescence-Assisted Transmigration Invasion and Motility Assay of ADSCs and PTX-ADSCs

ADSCs (50,000 cells/insert) were tagged with the lipophilic CellTracker CM-DiI dye (Invitrogen, Eugene, OR, USA) as described in [19]. Cells were then seeded in 150 µL serum-free medium in the upper side of fibronectin-coated (20 µg/mL) Boyden chambers, while chemoattractants were placed in the well below; then, 700 µL of complete medium (control), conditioned medium from SKOV3 (SKOV3-CM), SKOV3-MCTSs (SKOV3-MCTS-CM) or a cell layer of SKOV3 was used as the chemoattractant. Transmigrated cells were revealed using a computer-interfaced GeniusPlus microplate reader (Tecan).

### 2.6. Cocultivation of ADSCs with Ovarian Cancer Cells in Two Dimensional (2D) and Three Dimensional (3D) (Heterospherical) Models

OvCa cells (1.0 × 10^3^) were cultured in 96 well plates together with increasing number (0–1000) of ADSCs or PTX-ADSCs. After 7 days, cell viability was evaluated by MTT assay (2D model).

To obtain multicellular tumor spheroids (MCTSs) (3D model) SKOV3 cells (1 × 10^4^) were cultured in 24 well plates in non-adherent conditions (polyhema-coated wells). To monitor the self-assembly of ADSCs with SKOV3-MCTSs toward forming heterospheroids, ADSCs were stained before they were placed in cocultivation in non-adherent conditions with SKOV3-MCTSs (Supplemental Methods). To evaluate PTX-ADSCs antitumor activity in heterospheroids, SKOV3-MCTSs (1.0 × 10^4^ cells were cultured in 24 well plates in non-adherent conditions together with increasing number (0–1.0 × 10^4^) of ADSCs and PTX-ADSCs (heterospheroids). After 72 h heterospheroids were trypsinized to obtain a single cell suspension and cell viability was evaluated by Annexin-V (Becton-Dickinson Pharmingen) and propidium iodide (PI) staining, as previously described [20].

### 2.7. Cultivation of Ovarian Cancer Cells as a 2D Model in the Presence of ADSC-CM

Cell lines (1.0 × 10^3^ cells/well) were seeded in 96-well flat-bottomed microplates in 100 µL complete medium. Cells were allowed to adhere for 24 h, and then cultured with increasing concentrations of the reference drugs PTX (0–50 ng/mL) (free PTX), equivalent amounts of PTX released in PTX-ADSC-CM (0–100% *v*/*v*) (100% of PTX-ADSC-CM corresponds to 20 ng/mL secreted PTX) and ADSC-CM. After 7 days, cell viability was assayed using the MTT assay. Each experiment was conducted in triplicate. The IC50 values were calculated using the CalcuSyn software [18]. IC50-fold decrease was calculated as the ratio of the IC50 for free PTX to that of PTX secreted in PTX-ADSC-CM.

### 2.8. Cultivation of Ovarian Cancer Cells as a 3D Model (MCTSs) in the Presence of ADSC-CM

For cultivation, 1 × 10^4^ MCTS ovarian cancer cells were plated into poly-HEMA-coated 48-well plates (non-adherent conditions) for 72 h in complete medium and cultured with increasing doses of free PTX (0–50 ng/mL) or of PTX-ADSC-CM (0–75%) for 7 days. Then viable cells were evaluated using PrestoBlue Cell Viability Reagent (Thermo Fisher Scientific, Frederick, MD, USA).

The antitumor activity of PTX released in PTX-ADSC-CM was compared to equivalent amounts of free PTX in preformed single MCTSs. Briefly, 1.0 × 10^3^ SKOV3 cells were dispensed into poly-HEMA coated round-bottom 96-well. After 3 days, formed MCTSs (1 MCTS/well) were treated with increasing concentrations of PTX or PTX-ADSC-CM. Spheroid size was measured at day 7 after drug treatment initiation using an inverted microscope (Eclipse TS/100, Nikon, Tokyo, Japan) with photomicrographic systems DS Camera Control Unit DS-L2. Spheroid volumes were calculated using the formula: (width 2 × length × 3.14)/6 [21].

### 2.9. Migration (2D) and Dissemination/Invasion Assay

Cell migration was assessed using the in vitro scratch assay. Briefly, cells were grown to confluence and then treated with free PTX and PTX-ADSC-CM (equivalent to 5 ng/mL free PTX). After 72 h, monolayers were washed twice with PBS, scraped with a pipette tip to create a “wound” in the monolayer and washed again. Culture medium with 2% FBS was added and cells were cultured for 48 h. Wounds were photo-graphed using an inverted microscope (EclipseTS/100, Nikon, Instruments Europe BV Amsterdam, Netherlands) at magnification 4×. Migration was assessed by measuring the covered area (in pixels) with ImageJ-NIH (National Institutes of Health) tool software after 24 and 48 h. Tumor spheroid-based migration assay: this assay was performed in 96-well plates pre-coated with 10 μg/mL collagen I (Sigma Aldrich) and blocked with BSA (1 mg/mL) for 2 h [22]. Pre-formed tumor spheroids from SKOV3 and OVCAR8 cells were transferred (1 spheroid/well) into 96-well plates in the absence or in the presence of free PTX, ADSC-CM and PTX-ADSC-CM (equivalent concentrations). Image analysis software was used to calculate the area covered by migrated cells. The extent of migration was determined using Adobe Photoshop by outlining the entire area of the dispersed cells [23]. The fold-change (increase) in covered area was calculated dividing the pixel area of the spheroid at 48 h by the pixel area at time 0.

### 2.10. Statistical Analyses

Statistical analysis was carried out using GraphPad Prism version 6.0 software (GraphPad, LJ, USA), using the most appropriate test, as specified in each figure. Two sets of data differences were analyzed by Student’s *t*-test. One-way ANOVA, followed by the Bonferroni correction, was used for multiple comparisons. One-way ANOVA, followed by Dunnett’s test, was used to compare each of a number of treatments with a single control. *p*-value < 0.05 was considered significant.

## 3. Results

### 3.1. The Effect of PTX Uptake on ADSCs’ Activities

**Figure 1:**
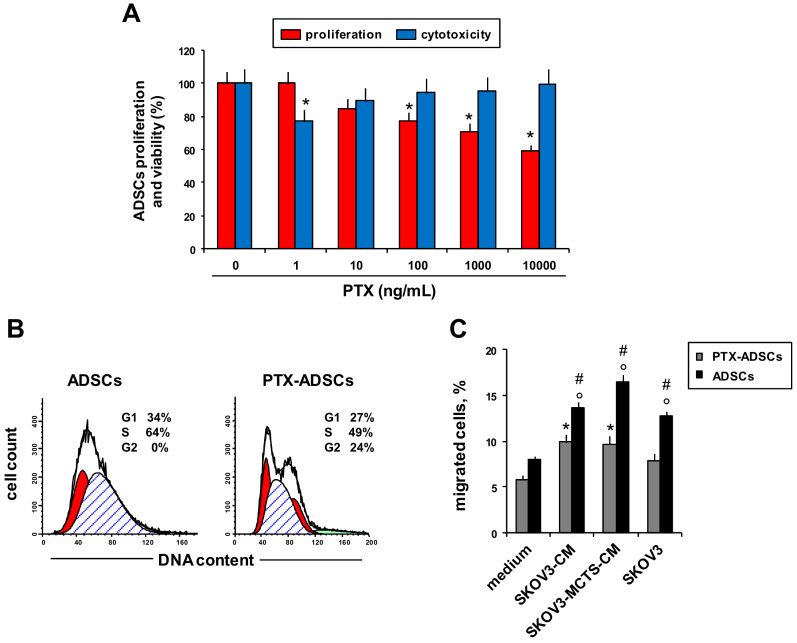
PTX-ADSCs’ viability, cell cycle phase distribution and migration. (**A**) ADSCs were cultured with PTX (0–10.000 ng/mL). Cell viability and proliferation were evaluated by MTT assay after 24 h and 7 d of treatment, respectively (percent of control). * *p* < 0.05 vs. no PTX, one-way ANOVA and Dunnett’s test. (**B**) Representative cytofluorimetric histograms showing cell cycle progression of ADSCs and PTX-ADSCs stained with propidium iodide. Results are means and SDs of three experiments. (**C**) Migration of ADSCs and PTX-ADSCs from fibronectin-coated upper chambers towards complete medium, SKOV3-CM, SKOV3-MCTS-CM and SKOV3 cells. Results are means and SDs of three experiments. * *p* < 0.05 vs. medium (ADSCs), ° *p* < 0.05 vs. medium (PTX-ADSCs), # *p* < 0.05 PTX-ADSCs vs. ADSCs one-way ANOVA and Bonferroni correction.

We evaluated PTX’s effects on ADSCs viability (Figure 1A, blue bar charts), proliferation (Figure 1A, red bar charts), cell cycle phase distribution (Figure 1B) and migration towards OvCa and CM from OvCa cells (Figure 1C).

We assessed the sensitivity of ADSCs to PTX in a 24 h cytotoxicity test and in an anti-proliferation assay after 7 d of treatment (Figure 1A). ADSCs had low sensitivity to the cytotoxic and antiproliferative effects of PTX (Figure 1A). A short incubation with PTX did not affect ADSCs viability and only the highest concentration of free-PTX (10 µg/mL) determined a reduction of proliferation of about 40% respect to control (untreated ADSCs) after 7 d of treatment (Figure 1A). ADSCs primed with PTX (2 µg/mL for 24 h) (PTX-ADSCs) secreted about 20 ng/mL of the drug (Supplementary Figure S1). PTX-ADSCs showed a slight decrease of the S phase and an increase in the G2/M phase of the cell cycle with respect to control ADSCs (Figure 1B). To evaluate migration of ADSCs after priming with PTX, we used as stimulus CM from SKOV3 cells (SKOV3-CM) or CM from SKOV3-MCTSs (SKOV3-MCTS-CM) and a layer of SKOV3 cells (Figure 1C). All chemoattractants, SKOV3 cells and SKOV3-CM (from SKOV3 layer and SKOV3-MCTSs), increased the percentage of migrated ADSCs with respect to the control (medium). Priming with PTX (PTX-ADSCs) reduced the capability of ADSCs to migrate towards SKOV3 cells, SKOV3-CM and SKOV3-MCTS-CM (Figure 1C).

### 3.2. PTX-ADSCs Inhibit OvCa Growth

**Figure 2:**
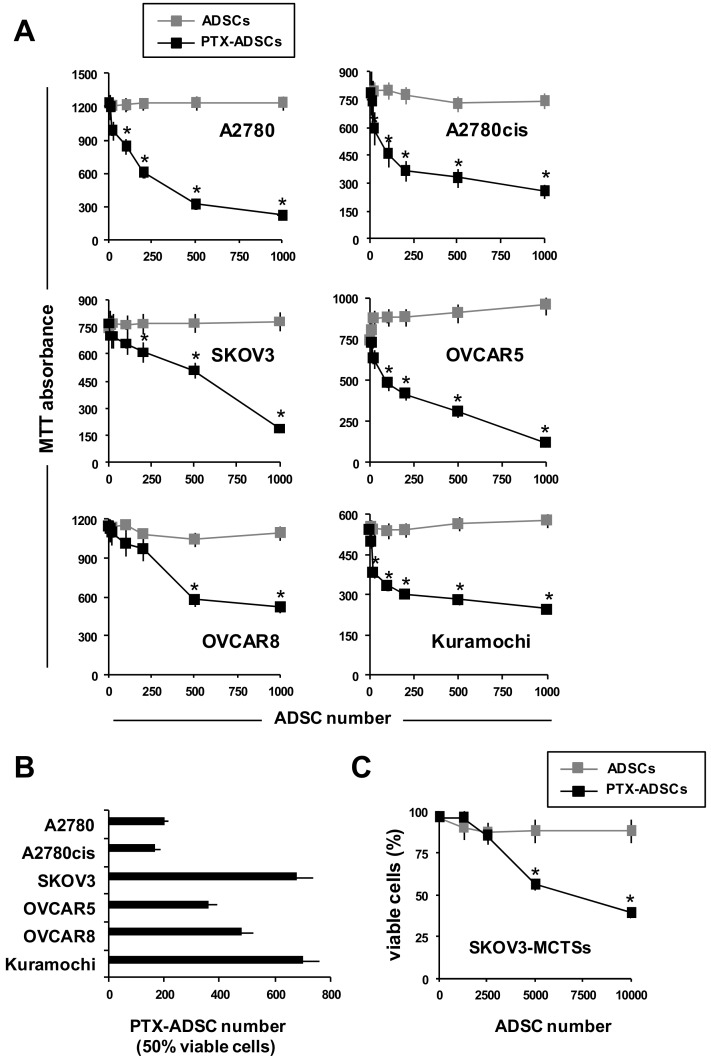
Cocultivation of OvCa cells with PTX-ADSCs kills in 2D and 3D tumor cells and overcomes PTX-resistance. (**A**) OvCa cells (1 × 10^3^) were cultured with increasing number of ADSCs and PTX-ADSCs (2D model). After 7 d, cell viability was evaluated by MTT assay (percent of control). * *p* < 0.05 vs. no ADSCs, one-way ANOVA and Dunnett’s test. (**B**) Number of PTX-ADSCs capable of reducing 50% of viable cells (MTT assay). Results are means and SDs of three experiments. (**C**) SKOV3-MCTSs were cultured under non-adherent conditions (3D model) with increasing numbers of ADSCs and PTX-ADSCs (heterospheroids). After 72 h cell viability was evaluated by AnnexinV/PI staining (% of control). Viable cells: AnnexinV/PI negative cells. Results represent the % of control (SKOV3-MCTSs) cultured without ADSCs. Results are means and SDs of three experiments. * *p* < 0.05 vs. no ADSCs, one-way ANOVA and Dunnett’s test.

To evaluate the anticancer activity of PTX-ADSCs, we cultured a fixed number of OvCa cells with increasing numbers of ADSCs and PTX-ADSCs (2D-direct contact) (Figure 2A). PTX-ADSCs decreased in a number-dependent manner tumor cell viability (Figure 2A). A2780 and its cisplatin-resistant clone A2780cis were the most sensitive to PTX secreted by PTX-ADSCs, as evaluated by the number of PTX-ADSCs capable of decreasing 50% of the cell viability (Figure 2B). On the contrary, ADSCs alone did not affect cancer cell viability of OvCa cells (Figure 2A). ADSCs added to SKOV3-MCTS under non-adherent conditions, aggregate to form heterospheroids (3D model) (Supplementary Figure S2).

The cytotoxic effects of PTX-ADSCs were confirmed in heterospheroids formed by SKOV3 cells and PTX-ADSCs. While the cultivation of SKOV3-MCTS cells with ADSCs did not affect cell viability, PTX-ADSCs (SKOV3/PTX-ADSCs heterospheroids) decreased in a number-dependent manner the percentage of viable cells in heterospheroids (Figure 2C).

### 3.3. PTX-ADSCs-CM is More Active than Free PTX and Overcomes PTX-Resistance in 2D Cultures

**Figure 3:**
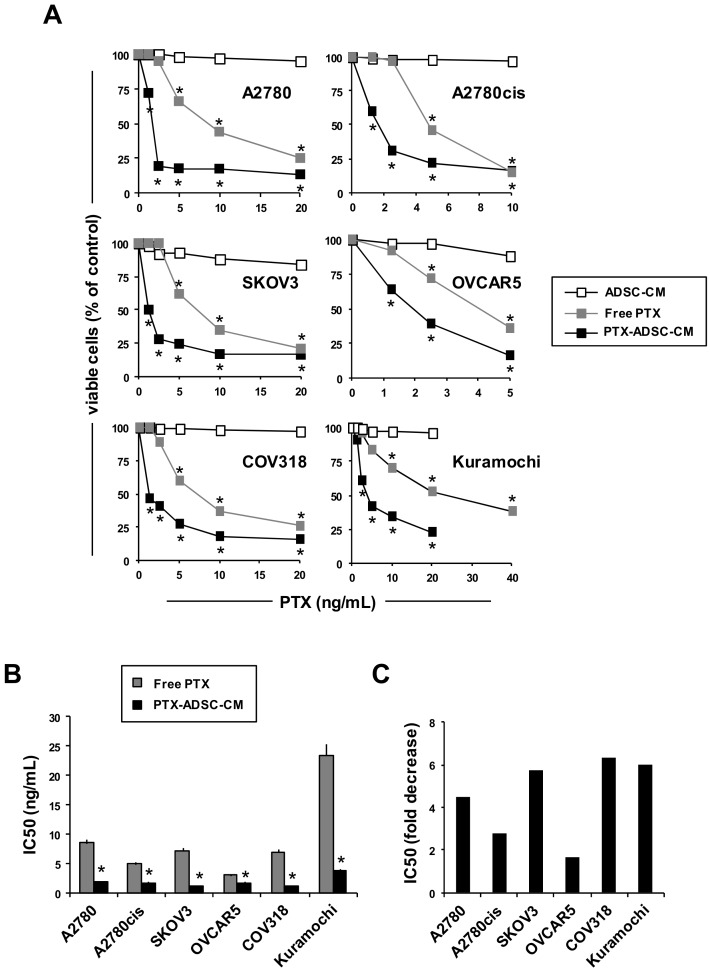
PTX-ADSC-CM kills OvCa cells, is more active than free PTX and overcomes PTX-resistance (2D model). (**A**) OvCa cells were cultured with ADSC-CM, PTX and equivalent amounts of PTX released by PTX-ADSCs in PTX-ADSC-CM (2D model). After 7 d of treatment, OvCa cell viability was evaluated by MTT assay. Results are means and SDs of three experiments. * *p* < 0.05 vs. no PTX, one-way ANOVA and Dunnett’s test. (**B**) IC50 (ng/mL) values for PTX and PTX in PTX-ADSC-CM calculated using CalcuSyn software. Results are means and SDs of three experiments. * *p* < 0.05 free PTX vs. PTX-ADSC, Student’s t test. (**C**) Fold decrease, calculated as the ratio of the IC50 for PTX to that of PTX secreted in ADSC-PTX-CM.

Then we compared free PTX cytotoxicity to PTX released in PTX-ADSC-CM (20 ng/mL) (Supplementary Figure S1) by testing different amounts of CM in parallel to the respective free PTX concentrations. Under these experimental conditions, ADSC-CM did not affect OvCa cell proliferation (Figure 3A), while both PTX-ADSC-CM and PTX decreased in a dose-dependent manner OvCa cell growth (Figure 3A). PTX-ADSC-CM (equivalent free PTX concentration) was more active than free PTX, with the lower IC50 (ranging from 1.1 to 3.9 ng/mL) than that obtained with free PTX (ranging from 3.0 to 23.3 ng mL) (Figure 3B).

PTX secreted by PTX-ADSCs counteracted PTX resistance in Kuramochi cells [15]. This cell line demonstrated an IC50 of 23.3 ng/mL for PTX that decreased to 3.9 ng/mL with PTX-ADSC-CM (equivalent to free PTX concentrations). Overall, PTX-ADSC-CM decreased the IC50 for PTX in all cell lines tested (Figure 3C) with fold decrease ranging from 1.6 (OVCAR5) to 6.3 (COV318) (Figure 3C).

### 3.4. PTX-ADSC-CM Inhibits OvCa Cell Migration

**Figure 4:**
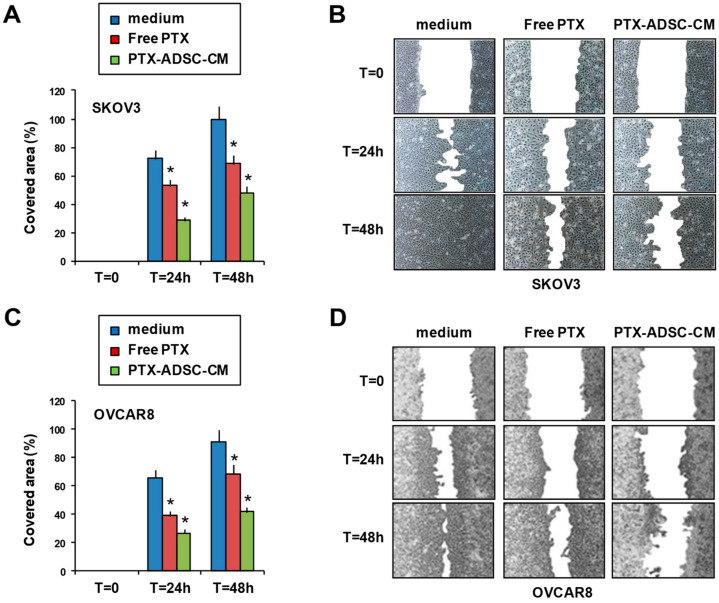
PTX-ADSC-CM reduces OvCa cell migration. Scratch assay. Confluent monolayers of SKOV3 (**A**,**B**) and OVCAR8 (**C**,**D**) cells were treated for 72 h in complete medium with PTX and PTX-ADSCs-CM (equivalent to 5 ng/mL of PTX), wounded by scraping and then cultured in low serum medium and photographed every 24 h. (**A**,**C**) Cell migration over time was expressed as cell covered area normalized to wound at time 0. Results are means and SDs of three experiments. * *p* < 0.05. vs. medium. (**B**,**D**) Representative phase contrast photomicrographs, original magnification 4×.

The effect of PTX-ADSC-CM on OvCa cell migration was evaluated using the in vitro scratch assay (2D model). Treatment with free PTX, and especially with PTX-ADSC-CM, slowed the ability of SKOV3 and OVCAR8 cells to refill an empty area (“scratch”) of the monolayer compared to untreated cells: 48 h after SKOV3 monolayer was scratched, the remaining uncovered area was about 52% in PTX-ADSC-CM, 32% in free PTX-pretreated cells, and almost totally covered in control cells (Figure 4A,B); in OVCAR8 the uncovered areas were 58%, 31.5%, and 9% for PTX-ADSC-CM, free PTX and medium, respectively (Figure 4C,D).

### 3.5. PTX-ADSC-CM is More Active than Free PTX in 3D MCTSs and Overcomes PTX Resistance

**Figure 5:**
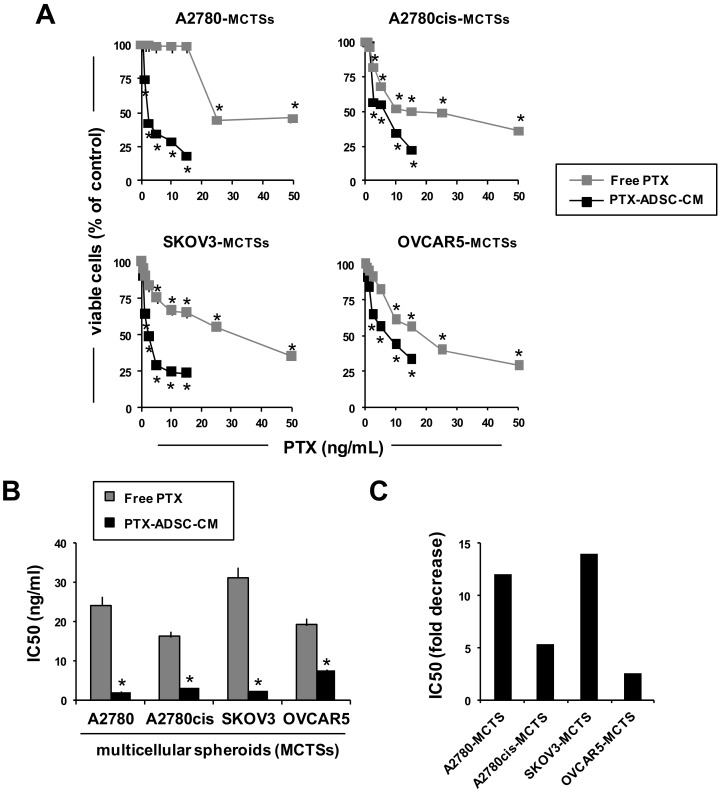
PTX-ADSC-CM kills OvCa-MCTSs, is more active than free PTX and overcomes PTX-resistance (3D model). (**A**) OvCa-MCTSs were cultured in non-adherent conditions with PTX and equivalent amounts of PTX released by PTX-ADSCs in PTX-ADSC-CM (3D model). After 7 d, OvCa-MCTSs viability was evaluated with Presto-Blue cell viability reagent. Results are means and SDs of three experiments. * *p* < 0.05. vs. medium, one-way ANOVA and Dunnett’s test. (**B**) IC50 (ng/mL) values for PTX and PTX in PTX-ADSC-CM calculated using CalcuSyn software. * *p* < 0.05 vs. free PTX, Student’s *t*-test. (**C**) Fold decrease, calculated as the ratio of the IC50 of PTX to that of PTX secreted in PTX-ADSC-CM.

We evaluated the cytotoxic effects of released PTX in PTX-ADSC-CM using MCTSs. Free PTX and PTX-ADCS-CM (equivalent concentrations) both inhibited OvCa MCTS cell viability (Figure 5A). Cultivation of OvCa cells as 3D MCTSs (non-adherent conditions) enhanced tumor cell resistance to PTX (IC50 ranging from 16 ng/mL to 31 ng/mL) (Figure 5A). This drug resistance due to aggregation in MCTSs was counteracted by PTX-ADSC-CM (IC50 ranged from 2 ng/mL to 7.5 ng/mL) (Figure 5B). Overall, PTX-ADSC-CM decreased the IC50 of PTX in all MCTSs tested with IC50-fold decrease (ratio of IC50 free PTX to IC50 PTX secreted in ADSC-PTX-CM) ranging from 2.5 (OVCAR5-MCTSs) to 14 (SKOV3-MCTSs) (Figure 5C).

**Figure 6:**
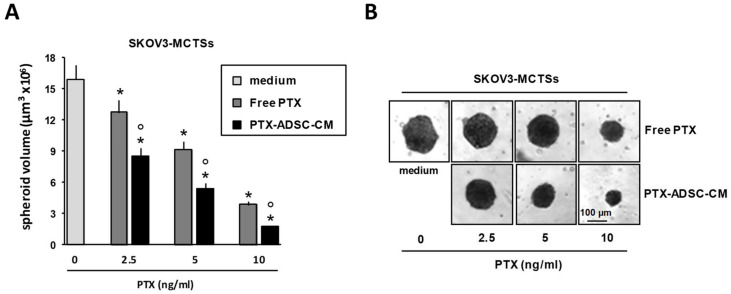
PTX-ADSC-CM kills OvCa-MCTSs and is more active than free PTX in MCTSs (3D model). (**A**) Three day old SKOV3 single pre-formed spheroids were cultured in the absence or in the presence of PTX and PTX-ADSC-CM (equivalent amounts of PTX). Responses were evaluated by spheroid volume measurements at day 7 after drug treatment. Values in the bar graph are means and SDs of five spheroids and three experiments. * *p* < 0.05 vs. medium, ° *p* < 0.05 vs. PTX, one-way ANOVA followed by the Bonferroni correction. (**B**) Representative phase contrast micro-photographs showing SKOV3-MCTS volume decrease by PTX and PTX-ADSC-CM treatment (original magnification 4×).

A2780, A2780cis and OVCAR5 formed small aggregates (spheroids) in non-adherent conditions, while the SKOV3 cell line formed large, dense aggregates (LDAs)—spheroids. Based on this observation, we used SKOV3 cells to quantify PTX activity in preformed MCTSs. PTX-ADSC-CM (equivalent concentrations) and free PTX both decreased SKOV3-MCTSs volume in size, but PTX in PTX-ADSC-CM was more efficacious than free PTX (Figure 6A,B).

### 3.6. PTX-ADSC-CM Inhibits OvCa MCTS Migration/Dissemination.

**Figure 7:**
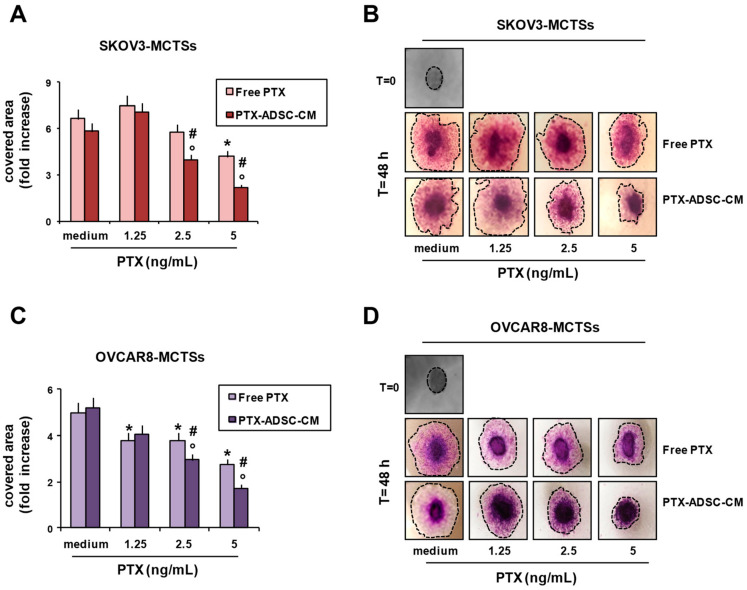
Inhibition of SKOV3-MCTS and OVCAR8-MCTS migration/dissemination onto matrix protein coating by PTX-ADSC-CM. (**A**–**D**) SKOV3 and OVCAR8 single spheroids were placed onto collagen-I coated plates in the presence of PTX (0–5 ng/mL) and PTX-ADSC-CM (equivalent amounts of PTX). After 48 h, OvCa cells were fixed with methanol and stained with crystal violet. (**A**,**C**) Bar charts showing the migration/dissemination rate of spheroids, evaluated as the area covered by migrating cells from spheroids and represented as fold increase respect to the area (pixel) covered at time = 0. Values in the bar graph represent the means ± SDs of three different experiments. * *p* < 0.05 free PTX vs. medium, ° *p* < 0.05 PTX-ADSC-CM vs. medium, # *p* < 0.05 PTX-ADSC-CM vs. free PTX, one-way ANOVA followed by Bonferroni correction. (**B**,**D**) Images were captured using an inverted microscope (phase contrast microphotographs, original magnification 4×). Dotted line indicates cell-covered area.

We evaluated the capability of SKOV3- and OVCAR8-MCTSs (forming LDA-spheroids) to migrate/disseminate onto collagen-coated wells. Treatment with free PTX and PTX-ADSC-CM inhibited SKOV3 (Figure 7A,B) and OVCAR8 (Figure 7C,D) migration, evaluated as the area covered by migrating cells. SKOV3-MCTS and OVCAR8-MCTS migrations were decreased by free PTX and to a greater extent by PTX released in PTX-ADSC-CM (Figure 7). ADSC-CM did not affect spheroid migration/dissemination (Supplementary Figure S3).

## 4. Discussion

Peritoneal carcinomatosis with formation of malignant ascites often characterizes the late stage of OvCa [12]. In ascitic fluid, exfoliated OvCa cells aggregate to form MCTSs and heterospheroids, composed by tumor cells and normal cells, including ADSCs [24,25]. Both MCTSs and heterospheroids in ascitic fluids are enriched in OvCa stem cells and contribute to drug resistance and spreading to secondary sites [24]. Thus, since MCTSs and heterospheroids mimic tumor growth in ascitic fluid and are considered effective first-line approaches to study in vitro drug activity, we used both cellular models to evaluate the anticancer effects of ADSCs primed with PTX (PTX-ADSCs).

Here, we found that the direct contact with PTX-ADSCs inhibited OvCa viability and overcame PTX resistance. Equivalent concentrations of PTX released by PTX-ADSCs were more cytotoxic than free PTX in 2D and 3D models (MCTSs) of OvCa; overcame intrinsic PTX resistance (Kuramochi cells); and overcame the resistance induced by culturing OvCa cells as MCTSs. Moreover, PTX released in PTX-ADSC-CM inhibited OvCa cell migration and dissemination of preformed MCTSs.

Primary debulking surgery followed by platinum and taxane chemotherapy is the standard treatment for advanced OvCa [12], but surgery can favor proliferation and invasion of residual cancer cells. We found that ADSCs primed with PTX released the drug and their cocultivation with OvCa cells was cytotoxic against both PTX and cisplatin resistant tumor cells. Thus, PTX-ADSCs used after surgery could counteract the proliferation/expansion of the residual drug-resistant cancer cells [24,26] and prevent the protective effects of ADSCs also in OvCa [25,26].

PTX-ADSCs maintained the capability to migrate towards molecules secreted by OvCa cells. PTX-ADSCs aggregated together with OvCa cells to form heterospheroids and inhibited both PTX and cisplatin-resistant MCTSs viability. These results suggest that PTX-ADSCs could be alternatively used during intraperitoneal chemotherapy (IP), where anticancer drugs are infused directly into the peritoneal cavity of patients, characterized by poor prognosis (stage III OvCa) and drug-resistance [12,27].

PTX efficacy is decreased by its low aqueous solubility and by multiple intrinsic or acquired drug resistance that can be mediated by different mechanisms, including the overexpression of the drug efflux transporter P-glycoprotein (Pgp) [28]. PTX released in CM from PTX-primed MSCs is secreted as free PTX and as PTX stored in EVs [5,6]. Our results suggest that PTX-ADSCs might provide PTX stored in EVs that, similarly to liposomal drugs, may cross plasma membranes more efficiently, and by increasing PTX accumulation, counteract Pgp-mediated efflux [29,30].

Most ovarian cancer patients’ present disseminated disease at the time of their diagnosis, which is one of the main reasons for their poor prognosis. OvCa cells shed from the primary tumor into the peritoneal cavity [31]; they have to survive to anoikis, a programmed cell death due to detachment from extracellular matrix [32] and immune surveillance [33]. Upon successful detachment from primary tumor, OvCa cells can survive by forming MCTSs or heterospheroids and then metastasize predominantly to the omentum and peritoneum via a direct mechanism [31,34]. Conditioned medium from ADSCs primed with PTX decreased, more efficiently than free PTX, OvCa cell 2D migration and also OvCa-MCTSs dissemination onto collagen gels, suggesting that PTX secreted by PTX-ADSCs may be more active in reducing metastasis formation as well.

In conclusion, both PTX-ADSCs and PTX-ADSC-CM decreased OvCa growth as MCTSs, and as heterospheroids, overcame the intrinsic PTX resistance of Kuramochi cells, the extrinsic resistance induced by aggregation of tumor cells as MCTSs and inhibited OvCa cell migration/dissemination. Results of our study suggest that both PTX-ADSCs and PTX-ADSC-CM may represent new natural systems, absent the necessity to engineer MSCs, with which to overcome intrinsic and extrinsic PTX resistance in OvCa, and potential new cancer therapies based on cell drug delivery.

## Supplementary Materials

The following are available online at https://www.mdpi.com/1999-4923/12/5/401/s1, Figure S1. LC-MS/MS quantification of taxol (PTX) released in PTX-ADSC-CM. Representative multi reaction monitoring (MRM) chromatogram for PTX quantification in control culture medium (A,A’) and released in ADSC-CM (B,B’). Red and blue lines: are referred to MRM-chromatogram for docetaxel used as internal standard (IS) and PTX respectively. The normalized PTX signal after. Figure S2. Heterospheroid formation. Time-Lapse Imaging of ADSC internalization in SKOV3 spheroids by Phase-Contrast and Fluorescence Microscopy. Representative photomicrographs of four-day-old SKOV3 spheroids cocultured with fluorescent DiI stained ADSC cells (ratio 1:1) (red). ADSC internalization was followed by time-lapse imaging using the Leica DMI6000 B Microscope for 96 h. Figure S3. Effects of ADSC-CM on OvCa MCTS migration/dissemination onto matrix protein coating. SKOV3 single spheroids were placed onto collagen-I coated plates in the presence of increasing concentrations (dilutions) of ADSC-CM. After 48 h, SKOV3 cells were fixed with methanol and stained with crystal violet. (A) Bar charts showing the migration/dissemination rate of spheroids, evaluated as the area covered by migrating cells from spheroids and represented as fold increase respect to the area (pixel) covered at time=0. Values in the bar graph represent the mean ± SD of three different experiments. (B) Images were captured using an inverted microscope (phase contrast microphotographs, original magnification 4x). Dotted line indicates cell-covered area. Numbers in the pictures indicate the fold increase of area covered by migrating cells respect to the area (pixel) covered at time = 0.
