# Supplementary material for: Adipose-Derived Stem Cells Primed with Paclitaxel Inhibit Ovarian Cancer Spheroid Growth and Overcome Paclitaxel Resistance
Source: Pharmaceutics. 2020 Apr 27;12(5):401. doi: 10.3390/pharmaceutics12050401 (PMC7284978; doi:10.3390/pharmaceutics12050401)
Supplement: Supplementary file 1 [file pharmaceutics-12-00401-s001.pdf]

pharmaceutics

Supplemental Materials: Adipose-Derived Stem Cells Primed with Paclitaxel Inhibit Ovarian Cancer Spheroid Growth and Overcome Paclitaxel Resistance

Cinzia Borghese, Naike Casagrande, Giuseppe Corona and Donatella Aldinucci

Methods

Paclitaxel analsyis in ADSC-CM

The concentration of paclitaxel (PTX) released in conditioned medium from ADSC/PTX (ADSC/PTX-CM) were measured using liquid chromatography coupled with tandem mass spectrometry (LC-MS/MS) according to Corona et al. [17] with few modifications. The analytical approach is based on an automatic on-line purification and enrichment technique followed by a measurement in tandem mass spectrometry through Multiple Reaction Monitoring (MRM). In brief, 50 μL of cell medium was added with 200 μL of acetonitrile solution containing docetaxel used as the internal standard (IS) (Fluka chemicals -Sigma-Aldrich Milan, Italy). After 5” vortex and centrifugation at 15,000*g* for 10 minutes at 4°C, the clear supernatant was transferred to auto-sample vial and introduced to the liquid chromatography system consisted of two Ultimate 3000 binary pumps and equipped with a 6-port switching valve (Dionex, Sunnyvale, CA-USA). On-line extraction step was performed through a 2.1 × 30 mm POROS-R1 perfusion column (Applied Biosystems, Foster City, CA-USA) while the chromatographic analytical step was performed by using a 3 × 50 mm, C18 Onyx column (Phenomenex, Tolerance CA-USA). The paclitaxel quantification was performed by the Hybrid mass spectrometer API 4000 Q-Trap connected online with the LC system (AB SCIEX, Toronto, Canada MA).The instrument was operated in positive ion mode with an ESI voltage of 5500 V. The collision energy was set to 22 and 12 eV for paclitaxel and for docetaxel internal standard, respectively. Declustering potentials were set at 52 V for paclitaxel and 40 V for docetaxel internal standard. The quantitative MRM transitions were m/z 854.1→286.1 for paclitaxel and m/z 808.2→527.0 for docetaxel (IS), with a 100 ms dwell time. The method was validated according to Food and Drug Administration guidelines. The low limit of quantification was 0.15 ng/mL, the linearity was in the range 0.15–1000 ng/mL, and the intra- and inter-assay accuracy and precision were less than 12%.

ADSC aggregation with SKOV3-MCTS (heteraspheroids formation)

Single spheroids were generated by culturing 1000 SKOV3 cells into poly-HEMA coated 96 round-bottomed wells for 4 days. Then ten spheroids were collected and cocultered with 1:1 ADSC (10^4 cells) that were previously stained with lipophilic CellTracker™ CM-DiI Dye (Thermo Fisher Scientific). Red-stained-ADSC were followed by time-lapse imaging using the Leica DMI6000 B Inverted Microscope for 96 h [20].

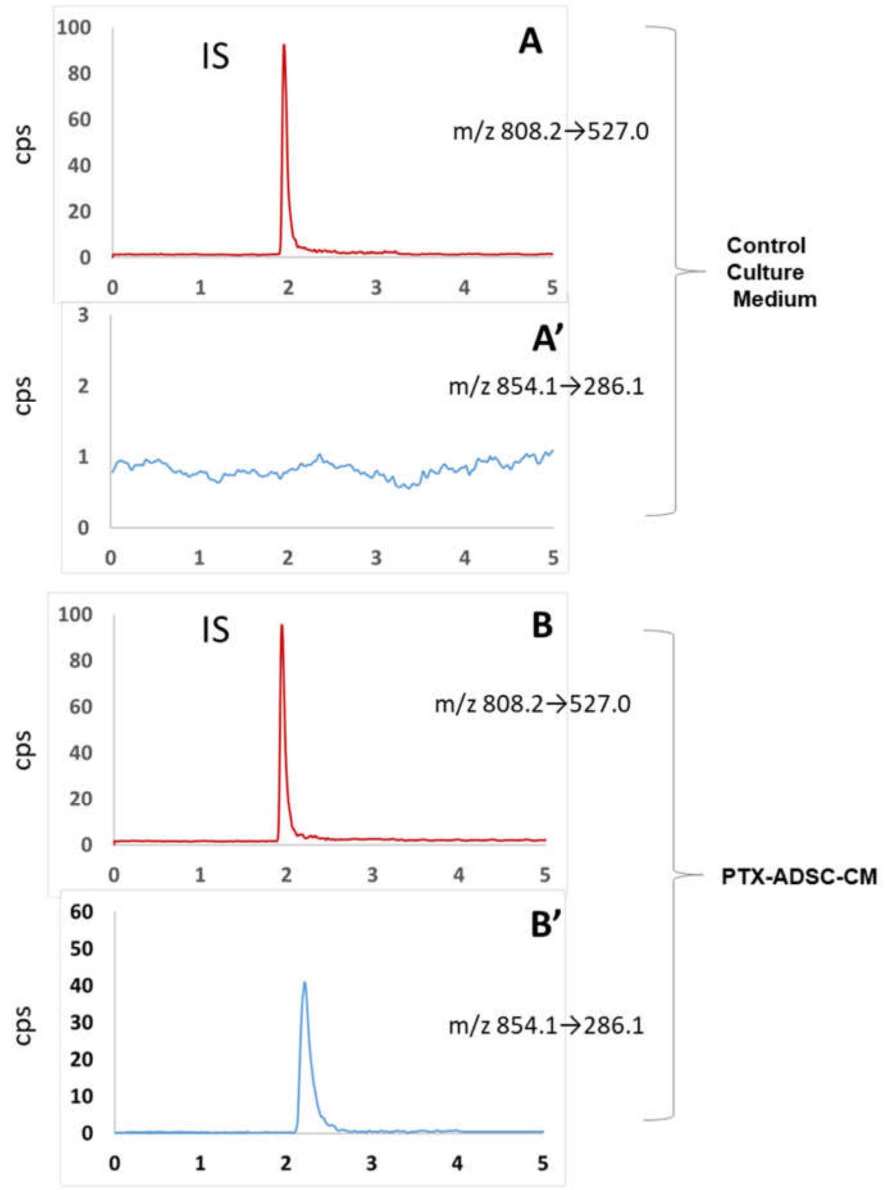

Figure S1. LC-MS/MS quantification of taxol (PTX) released in PTX-ADSC-CM. Representative multi reaction monitoring (MRM) chromatogram for PTX quantification in control culture medium (A,A’) and released in ADSC-CM (B,B’). Red and blue lines: are referred to MRM-chromatogram for docetaxel used as internal standard (IS) and PTX respectively. The normalized PTX signal after.

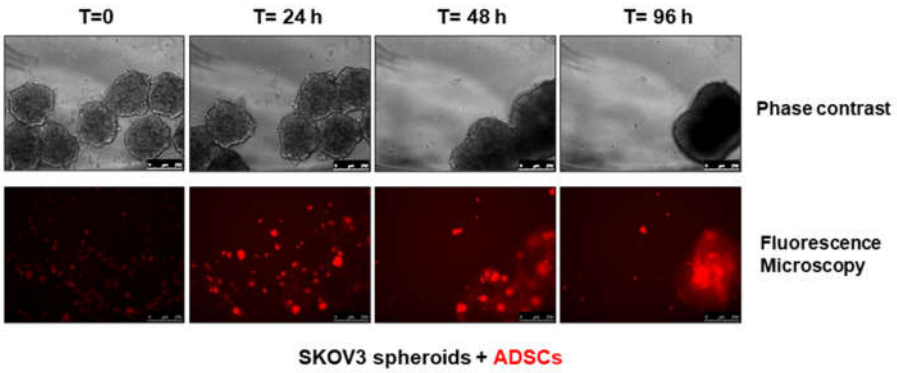

Figure S2. Heterospheroid formation. Time-Lapse Imaging of ADSC internalization in SKOV3 spheroids by Phase-Contrast and Fluorescence Microscopy. Representative photomicrographs of four-day-old SKOV3 spheroids cocultured with fluorescent DiI stained ADSC cells (ratio 1:1) (red). ADSC internalization was followed by time-lapse imaging using the Leica DMI6000 B Microscope for 96 h.

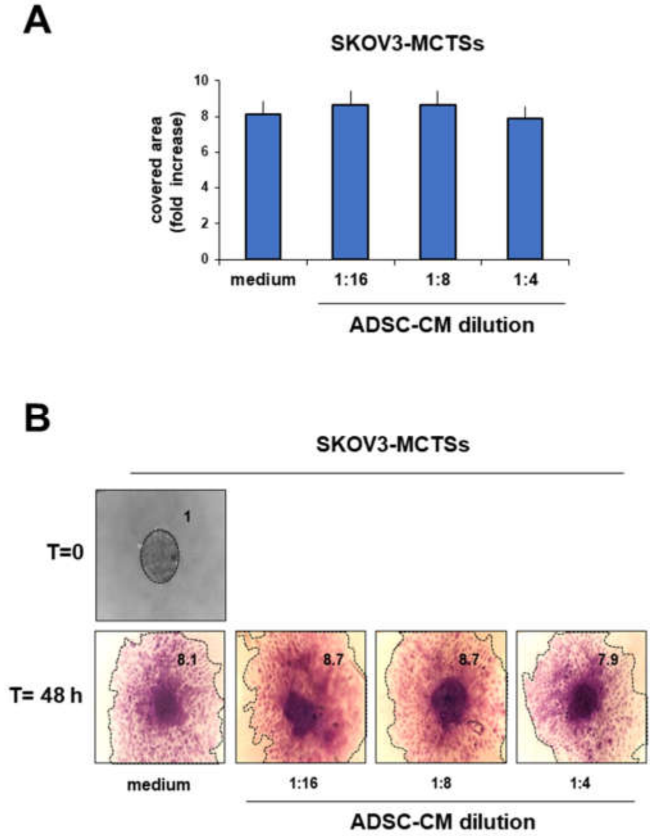

Figure S3. Effects of ADSC-CM on OvCa MCTS migration/dissemination onto matrix protein coating. SKOV3 single spheroids were placed onto collagen-I coated plates in the presence of increasing concentrations (dilutions) of ADSC-CM. After 48 h, SKOV3 cells were fixed with methanol and stained with crystal violet. (A) Bar charts showing the migration/dissemination rate of spheroids, evaluated as the area covered by migrating cells from spheroids and represented as fold increase respect to the area (pixel) covered at time=0. Values in the bar graph represent the mean ± SD of three different experiments. (B) Images were captured using an inverted microscope (phase contrast microphotographs, original magnification 4x). Dotted line indicates cell-covered area. Numbers in the pictures indicate the fold increase of area covered by migrating cells respect to the area (pixel) covered at time=0.
